# Machine‐learning‐based detection of adaptive divergence of the stream mayfly *Ephemera strigata* populations

**DOI:** 10.1002/ece3.6398

**Published:** 2020-06-15

**Authors:** Bin Li, Sakiko Yaegashi, Thaddeus M. Carvajal, Maribet Gamboa, Ming‐Chih Chiu, Zongming Ren, Kozo Watanabe

**Affiliations:** ^1^ Insititute of Environmental and Ecology Shandong Normal University Jinan China; ^2^ Department of Civil and Environmental Engineering Ehime University Matsuyama Japan; ^3^ Department of Civil and Environmental Engineering University of Yamanashi Yamanashi Japan

**Keywords:** adaptive divergence, altitude, aquatic insect, local adaptation, random forest, STRUCTURE

## Abstract

Adaptive divergence is a key mechanism shaping the genetic variation of natural populations. A central question linking ecology with evolutionary biology is how spatial environmental heterogeneity can lead to adaptive divergence among local populations within a species. In this study, using a genome scan approach to detect candidate loci under selection, we examined adaptive divergence of the stream mayfly *Ephemera strigata* in the Natori River Basin in northeastern Japan. We applied a new machine‐learning method (i.e., random forest) besides traditional distance‐based redundancy analysis (dbRDA) to examine relationships between environmental factors and adaptive divergence at non‐neutral loci. Spatial autocorrelation analysis based on neutral loci was employed to examine the dispersal ability of this species. We conclude the following: (a) *E. strigata* show altitudinal adaptive divergence among the populations in the Natori River Basin; (b) random forest showed higher resolution for detecting adaptive divergence than traditional statistical analysis; and (c) separating all markers into neutral and non‐neutral loci could provide full insight into parameters such as genetic diversity, local adaptation, and dispersal ability.

## Graphical abstract

In this study, using a genome scan approach to detect candidate loci under selection, we detected altitudinal adaptive divergence among the populations of the stream mayfly *Ephemera strigata* in the Natori River Basin in northeastern Japan. The applied machine‐learning method (i.e., random forest) showed high higher resolution for detecting adaptive divergence than traditional statistical analysis.

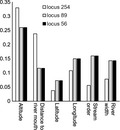

## 1 INTRODUCTION

A central question linking ecology with evolutionary biology is how spatial environmental heterogeneity can lead to adaptive divergence among local populations within a species. In stream ecosystems, adaptive divergence of aquatic insects is usually reported to be influenced by altitudinal gradient at the river corridor scale (Hughes, Schmidt, & Finn, 2009; Keller, Alexander, Holderegger, & Edwards, 2013; Polato et al., 2017). The underlying mechanism is that altitude is often strongly related to a number of environmental factors such as temperature and oxygen availability which greatly influenced the life of organisms (Lytle & Poff, 2004; Halbritter, Billeter, Edwards, & Alexander, 2015; Keller & Seehausen, 2012). Thermal regimes directly regulate the growth of species, development, and mating behavior, and setting limits on distributions and abundances of species across landscapes (Li et al., 2013). Oxygen availability also restricts distributions by affecting respiratory metabolism of aquatic organisms (Rostgaard & Jacobsen, 2005).

Recently, there has been an increase in studies on the genetic basis of adaptive divergence in aquatic insects because of their important role in freshwater ecosystem biomonitoring. Altitudinal genetic divergence has been reported in aquatic insects including caddis flies:*Plectrocnemia conspersa* and *Polycentropus flavomaculatus* (Wilcock, Bruford, Nichols, & Hildrew, 2007); *Stenopsyche maramorata* (Yaegashi, Watanabe, Monaghan, & Omura, 2014); stone flies:*Dinocras cephalotes* (Elbrecht et al., 2014); and mayflies:*Atalophlebia* (Baggiano, Schmidt, Sheldon, & Hughes, 2011). However, most of these studies were based on a given gene or a limited number of candidate genes.

The development of genome scanning approaches, such as amplified fragment length polymorphism (AFLP), allows the study of numerous anonymous markers (loci) rather than the study of a few candidate genes. Compared with neutral loci, loci influenced by directional selection (i.e., non‐neutral loci) are expected to exhibit higher levels of genetic divergence (Kirk & Freeland, 2011). Therefore, by screening large numbers of candidate loci (“outlier” loci, reviewed by Nosil, Funk, & Ortiz‐Barrientos, 2009), statistical methods can identify loci that are under direct selection or linked to loci under selection based on the level of genetic divergence. Selected non‐neutral loci can be used to test hypotheses about the adaptive process. Also, neutral loci may be available for accurate tests of neutral processes, such as isolation by distance (IBD) (Oleksa, Chybicki, Gawroński, Svensson, & Burczyk, 2013) and gene flow patterns, avoiding the confounding effects of natural selection (Kirk & Freeland, 2011).

In the ordinary analysis of genome scanning, non‐neutral loci are detected based on genetic variation among populations with different phenotypes or ecotypes (Bonin, Taberlet, Miaud, & Pompanon, 2006; Egan, Nosil, & Funk, 2008; Galindo & Rolán‐Alvarez, 2009; Nosil, Egan, & Funk, 2008) or allopatric populations among different geographic localities (Gaggiotti et al., 2009; Medugorac et al., 2009; Renaut, Nolte, Rogers, Derome, & Bernatchez, 2011). Genome scanning can also be conducted using genetically defined populations with unknown phenotypes or ecotypes. For example, Bayesian clustering methods (Falush, Stephens, & Pritchard, 2003, 2007; Pritchard, Stephens, & Donnelly, 2000) can delineate genetic populations prior to any observable phenotypic divergence and, therefore, may provide insights into the early stages of adaptive divergence (Whiteley et al., 2011).

Determining the link between non‐neutral loci and environmental factors is one of the most difficult tasks in molecular ecology. Conventional statistical methods such as the partial Mantel test (Legendre & Fortin, 2010; Watanabe, Kazama, Omura, & Monaghan, 2014), distance‐based redundancy analysis (dbRDA) (Watanabe & Monaghan, 2017), and multivariate analysis of variance (MANOVA) (Mccairns & Bernatchez, 2008) have been widely applied. However, these methods pose certain issues and limitations. One issue is the tendency of bias and high error rates that result from associating genetic variance and environmental distances (Guillot & Rousset, 2013; Legendre & Fortin, 2010; Legendre, Fortin, & Borcard, 2015). In addition, the Mantel test and dbRDA are limited to testing the linear independence between genetic and environmental distances among local populations. This may due to the nonlinearity of these distances and possible information loss in the converting process. Additionally, there is often much difficulty in fulfilling underlying assumptions (e.g., normal distribution and homogeneity of variance) of conventional statistical methods such as MANOVA or multiple linear regression (Vittinghoff, Glidden, & Mcculloch, 2012). Because of these concerns, modern statistical techniques, such as machine‐learning methods, are now being developed as promising alternatives. Machine‐learning methods are particularly effective in finding and describing structural patterns in data and providing the values of relative importance among variables (Biau & Scornet, 2016; Prasad, Iverson, & Liaw, 2006).

Among the variety of machine‐learning methods available, random forest (RF) (Breiman, 2001) is one of the most widely used modeling techniques to generate high prediction accuracy and evaluate the relative importance of explanatory variables in the model (Biau & Scornet, 2016). RF is an ensemble tree‐based method that constructs multiple decision trees from a data set and combines results from all the trees to create a final predictive model. In ecological studies, RF has been applied to community‐level studies to predict the distributions of species and identify constrained environmental factors (Evans, Murphy, Holden, & Cushman, 2011; Pelletier, Carstens, Tank, Sullivan, & Espíndola, 2018; Smith & Carstens, 2019; Wedger, Topp, & Olsen, 2019). In most of these studies, environmental data have been used as independent variables to predict the presence or absence of species (dependent variables). The relative contributions of environmental variables to the distribution of species are quantified by their relative importance obtained from the RF model. It may therefore be possible to extend the use of RF to population genetic studies where environmental variables are used to predict the presence or absence of haplotypes or alleles at outlier loci. The relative importance of each environmental variable could be considered as its influence to outlier loci, which may strongly drive adaptive divergence.

In this study, we examined adaptive divergence using AFLP markers in populations of the stream mayfly *Ephemera strigata* from the Natori River Basin in northeastern Honshu Island, Japan. We have two main objectives: The first is to determine the extent of local adaptation at the genome level in natural populations and to quantify associations between environmental gradients and adaptive divergence, and the second objective is to apply a modified machine‐learning method for determining the selection pressure on outlier loci. We first detected loci under selection (non‐neutral loci) based on locus‐specific genetic differentiation among populations. Rather than defining populations a priori using geographic or phenotypic information, we delineated populations based on the discontinuities in the AFLP variation among individuals using a hierarchical analysis of STRUCTURE (Falush, Stephens, & Pritchard, 2003, 2007; Pritchard et al., 2000; Vähä, Erkinaro, Niemelä, & Primmer, 2007). Secondly, focusing on non‐neutral loci, we employed a machine‐learning method (i.e., RF) to identify environmental variables most likely to contribute to adaptive divergence. Additionally, we also conducted the ordinary distance‐based redundancy analysis (dbRDA) to comparatively examine the feasibility of the method. Finally, focusing on the neutral loci, we examined dispersal pattern and dispersal distance of the species.

## 2 METHODS

### 2.1 Study site and sampling

**FIGURE 1:**
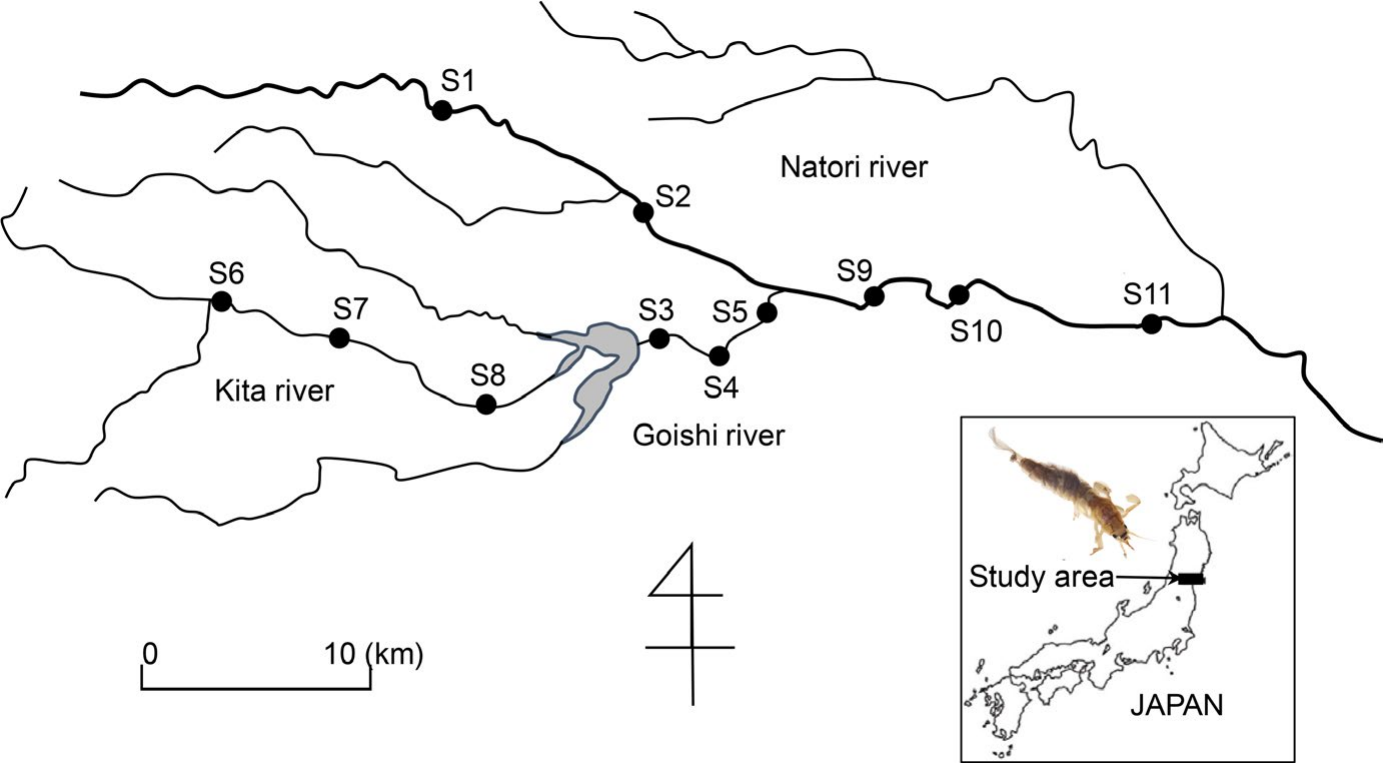
Map of 11 sampling sites and photograph of species *Ephemera strigata* in the Natori River Basin in northeastern Japan

*Ephemera strigata* is a well‐studied mountain burrowing mayfly in Japan and Korea (Ban & Kawai, 1986; Lee, Hwang, & Bae, 2008). In this study, sampling was carried out in the Natori River catchment in the Miyagi Prefecture in northeastern Japan (Figure 1). Nymphal samples were collected at 11 sites from October 26 to November 12, 2010. At each site, we collected *E. strigata* individuals using sa Surber net (30 × 30 cm quadrat with mesh size 250 µm) along 200–900 m stream reaches. All specimens were preserved in the field in 99.5% ethanol, transported to the laboratory, and identified to species level under a stereomicroscope (120×) using taxonomic keys (Kawai & Tanida, 2005).

We measured seven geographic parameters at each site using standard ecological methods in stream surveys (Hauer & Lamberti, 2007; Watanabe, Monaghan, & Omura, 2008). Stream order was determined using a 1:25,000 map. The width of the stream channel was measured as average value at 10 randomly selected cross sections using a tape measure. Longitude and latitude coordinates and altitude were recorded using a global positioning system on the riverside. Distance to river mouth was the distance between sampling site and river mouth, and riverine distance was the river course distance between each pair of two sites. Both parameters were measured on Google Maps using the ruler function. Because there is no correlation among selected variables based on collinearity analysis (variance inflation factor [VIF] < 10), we included all those variables in our further analysis.

### 2.2 DNA extraction and AFLP fingerprinting

DNA extraction was performed on abdominal tissues after digestive tract removal. The DNA of each individual was extracted using the DNeasy 96 Blood & Tissue Kits (Qiagen). The concentration of extracted DNA was measured on a NanoDrop ND 1000 spectrometer (Thermo Fisher Scientific) and diluted to 50 ng/µl.

We genotyped 216 individuals from 11 sites with the AFLP method (Vos et al., 1995). The restriction step followed the protocol by Watanabe et al. (2014). The ligation step was performed by adding 1 U T4 DNA ligase (New England), 0.2 µl of 100 µM MseI adapter, 0.2 µM of EcoRI adapter, 2 µl T4 DNA ligase buffer (10×) (New England), and up to 20 µl dH_2_O and incubating the solution at 16°C for 12 hr. The sequences of the MseI adapter and EcoRI adapters were extracted from Reisch (2007). The adapters were manually prepared as follows: (a) mixing equal molar amounts of adapter oligomer, (b) denaturing at 95°C for 5 min, and (c) incubating for 10 min at room temperature. Restricted or ligated products were then diluted at a 1:19 ratio with 0.1 × TE buffer. Preselective amplification was performed in a mixture of 0.06 µl of 100 µM MseI and EcoRI primers (Reisch, 2007), 15 µl of AFLP Amplification Core Mix (Applied Biosystems), 4 µl of each restricted/ligated product, and up to 29 µl dH_2_O. Preselective polymerase chain reaction (PCR) parameters followed Reisch (2007). PCR products were diluted 20 times by 0.1 × TE buffer.

For selective amplifications, we employed three types of primer pairs (EcoRI‐AGG & MseI‐CAT, EcoRI‐ACC & MseI‐CAC, and EcoRI‐AGG & MseI‐CAC) that generate the most variable patterns in 64 types of selective primer pairs using three individuals. Each EcoRI primer was modified with Beckman Dye 2, 3, or 4 on the 5′‐end. The mixture of selective PCR was 0.1 µl of 100 µM MseI and EcoRI primers, 15 µl of AFLP Amplification Core Mix (Applied Biosystems), and up to 20 µl dH_2_O. We followed Reisch (2007) to set PCR parameters.

The selective PCR products were separated by capillary gel electrophoresis using CEQ8000 (Beckman Coulter). We extract all fragments which were ranged from 60 bp to 360 bp, using the default parameters. To adjust fluorescent intensity, each fluorescent PCR product was mixed with the following: EcoRI‐AGG & MseI‐CAT 4 µl, EcoRI‐ACC & MseI‐CAC 2 µl, and EcoRI‐AGG & MseI‐CAC 1 µl. Peak sizes of PCR products were calibrated with DNA Size Standard 600 (Beckman Coulter) and calculated using the CEQ8000 software (Beckman Coulter) as per the instruction of manufacturer.

### 2.3 Hierarchal STRUCTURE analysis

We defined populations based on discontinuities in AFLP variation using the individual‐based Bayesian clustering method implemented in STRUCTURE v. 2.3 (Falush et al., 2003, 2007; Pritchard et al., 2000). We performed 20 runs of 50,000 iterations with a burn‐in of 10,000 for each number of assumed populations (*K*) ranging from 1 to 15 using the admixture model and assuming correlated allele frequencies. We used a uniform prior for alpha (the parameter representing the degree of admixture) with a maximum of 10 and set Alphapropsd to 0.05. Lambda, the parameter representing the correlation in the parental allele frequencies, was estimated in a preliminary run using *K* = 1. The prior *F*_ST_ was set to the default value (mean = 0.01; standard deviation (*SD*) = 0.05).

To determine the optimal *K*, we computed the log‐likelihood (Ln P (*K*)) for each *K* and selected *K* with the highest standardized second‐order rate of change (∆*K*) of Ln P (*K*) (Evanno, Regnaut, & Goudet, 2005). Although this method helps to correctly identify *K* in most situations, it is known to have two limitations. First, it is useful only for the uppermost level of a hierarchical genetic structure. Second, it is unable to find the best *K* if *K* = 1 (i.e., if there is no population substructure) (Evanno et al., 2005). To address these limitations, we used a hierarchical approach for STRUCTURE analysis modified from Vähä et al. (2007), which repeats the analysis at lower hierarchical levels until no substructure can be uncovered. The advantage of our method was that we used the Wilcoxon two‐sample test to control the round of repeated analysis instead of checking the pattern of individual membership. Specifically, we compared the mean value of Ln P (*K*) from 20 runs with the optimal *K* (as determined using ∆*K*) with mean Ln P (*K* = 1) using the Wilcoxon two‐sample test (Rosenberg et al., 2001). If Ln P (*K* = 1) was found to be significantly lower than Ln P (*K*) at the optimal *K*, we repeated the analysis within each of the *K* populations. At each hierarchical level, individuals were assigned to subpopulations based on the individual membership coefficient (Pritchard et al., 2000).

### 2.4 Outlier loci detection

We used two different statistical methods to identify outlier loci. Dfdist (adapted from Fdist; Beaumont & Nichols, 1996) uses coalescent simulations to generate thousands of loci evolving under a neutral model of symmetrical islands with a mean global *F*_ST_ close to the observed global *F*_ST_. Mean *F*_ST_ was calculated using the default method by first excluding 30% of the highest and lowest observed values. Empirical loci with *F*_ST_ values significantly greater (*p* < .05) than the simulated distribution (generated with 50,000 loci) were considered to be outliers. Dfdist can detect both divergent selection and balancing selection; however, we focused only on divergent selection in this study. BayeScan is a hierarchical Bayesian model‐based method first described in Beaumont and Balding (2004) and modified by Foll and Gaggiotti (2008) for dominant markers (available at http://cmpg.unibe.ch/software/bayescan/). This Bayesian method is based on the concept that *F*_ST_ values reflect contributions from locus‐specific effects, such as selection, and population‐specific effects, such as local effective size and immigration rates. The main advantage of this approach is that it allows for different demographic scenarios and different amounts of genetic drift in each population (Foll & Gaggiotti, 2006, 2008). Using a reversible jump Markov chain Monte Carlo approach, the posterior probability of each locus being subjected to selection is estimated. A locus is deemed to be influenced by selection if its *F*_ST_ is significantly higher or lower than the expectation provided by the coalescent simulations. For all subsequent analyses, non‐neutral loci were defined as outlier loci detected by the Dfdist and BayeScan methods at the 95% confidence level. Neutral loci were defined as loci detected by neither Dfdist nor BayeScan at the 95% thresholds. Loci detected as outliers by only one of the two methods were not considered in the further analyses. In order to check whether there have some loci being misidentified as outlier due to linkage disequilibrium, we further tested for pairwise linkage disequilibrium (LD) of the outlier loci detected by both methods, using 1,000 steps in the Markov chain and a dememorization of 1,000 steps in ARLEQUIN 3.5 (Excoffier & Lischer, 2010).

### 2.5 Analysis of genetic diversity

*F*_ST_ was calculated with ARLEQUIN v. 3.5 using (a) all loci, (b) only neutral loci, and (c) only non‐neutral loci. Global heterozygosity among all populations (*H_t_*) and mean heterozygosity within populations (*H_w_*) were estimated separately for neutral and non‐neutral loci with AFLP‐SURV v. 1.0 (Vekemans, Beauwens, Lemaire, & Roldán‐Ruiz, 2002) using the Bayesian method with a uniform prior distribution of allele frequencies (Zhivotovsky, 1999). Molecular variance analysis (AMOVA) was also conducted using ARLEQUIN to provide the estimates of genetic variations among and within sampling sites. For the test of IBD, we examined the correlations of pairwise *F*_ST_ with geographic distance and riverine distance (i.e., distance along the watercourse) between sites using GeneAlEx v. 6.5 (Peakall & Smouse, 2012). The genetic distance between each pair of sites was quantified using mean pairwise *F*_ST_ for neutral and non‐neutral loci using the Bayesian‐estimated allele frequencies generated by AFLP‐SURV.

We conducted genetic spatial autocorrelation analysis using neutral loci for geographic distance. Eight geographic distance classes defined every 4 km (from 0–4 km to 28–32 km) were used in the analysis. Individuals within the same site were considered to be separated by a distance of 0 km. We calculated Moran's I for each distance class using GeneAlEx, where I ranges from −1 to 1 and the positive values indicate that sites within a given distance class have similar genetic structure. We used jackknifing to estimate the 95% confidence intervals.

### 2.6 Adaptive divergence modeling

We determined the environmental variables that drive adaptive divergence at non‐neutral loci using the RF model (Blagus & Lusa, 2013; Chawla, Bowyer, Hall, & Kegelmeyer, 2002; Maciejewski & Stefanowski, 2011). Stream order, width of the stream, longitude, latitude, altitude, and distance to river mouth were used to predict the band presence/absence patterns at each non‐neutral locus for each individual. We assigned individuals from the same site to the same environmental conditions. The data set was imbalanced because the number of individuals with band presence was not equal to that with band absence. The individuals were thus classified into two classes (i.e., presence and absence). We solved the data imbalance problem by oversampling for the minority class through the synthetic minority oversampling technique (SMOTE) (Chawla et al., 2002) using SMOTE function in DMwR package (Torgo, 2013). SMOTE creates synthetic minority class sample units by taking the difference between the feature vector (sample) under consideration and its nearest neighbor. It then multiplies this difference by a random number between 0 and 1 and adds it to the feature vector under consideration (Chawla et al., 2002). The RF model for each non‐neutral locus was built using randomForest function in randomForest package (Liaw & Wiener, 2002) in the R program (R Development Core Team, 2015). Model performance was evaluated using the area under the receiver operating characteristic curve (AUC) (Janitza, Strobl, & Boulsesteix, 2013). The AUC value typically ranged from 0.5 (random prediction) to a maximum value of 1, which represents the theoretical perfect model. As rules of thumb, an AUC value greater than 0.9 indicates very good model quality, a value smaller than 0.7 indicates poor model quality, and a value between 0.7 and 0.9 indicates good model quality (Baldwin, 2009).

We also conducted dbRDA as a comparative ordinary method. Among the seven environmental variables, we searched for the variables that best explain the most variation in F_ST_ at non‐neutral loci. DbRDA was performed on the ordination solutions, rather than on the distance matrices (Legendre & Fortin, 2010). In this study, pairwise genetic distances at non‐neutral loci among sites were used to screen environmental factors that most closely relate to genetic divergence (Watanabe & Monaghan, 2017). The best model, comprising significant predictors, was selected using forward selection with permutation tests and an inclusion threshold of *α* = 0.05 using the ordistep function of the vegan package (Oksanen et al., 2018) in the R program (R Development Core Team, 2015). Significant differences were tested with the ANOVA.cca function in the vegan package.

## 3 RESULTS

### 3.1 Hierarchical STRUCTURE analysis

**FIGURE 2:**
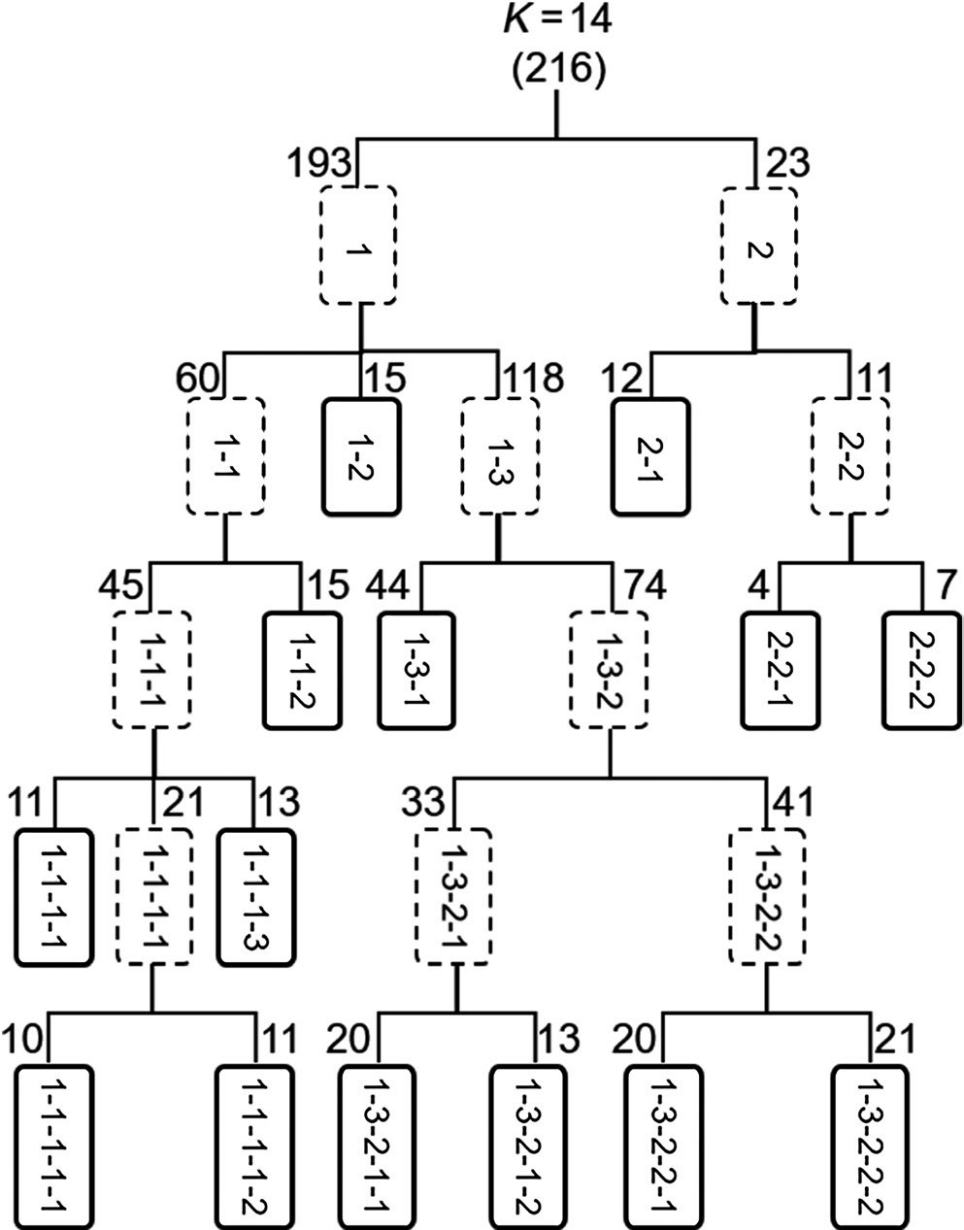
Subpopulation structure of *Ephemera strigata* as determined using STRUCTURE with hierarchical iterations. Dashed boxes indicate subpopulations, and solid boxes indicate final populations. Numbers at the top of boxes indicate the number of individuals assigned to the populations. A total of 14 groups (*K*) were defined from 216 individuals

Hierarchical iterations by STRUCTURE detected significant substructure until the 4th iteration beyond the initial analysis (Figure 2). A total of 14 groups were defined for the 216 *E. strigata* individuals collected in 11 sites. The numbers of individuals assigned in each group were ranged from 4 to 44 (mean = 15.4; *SD* = 9.5). Most groups were widespread all over the sampling sites, whereas some groups were restricted to specific sites (data not shown). For example, the members of groups 2, 3, and 8 occurred only in upstream and middle‐stream sites (Figure 1: upstream sites, S1 and S6‐8; middle‐stream sites, S2‐5).

### 3.2 Outlier detection and genetic diversity

Using our criterion of 95% significance with both Dfdist and BayeScan, 10 non‐neutral loci and 346 neutral loci were detected from the 372 polymorphic AFLP loci. Dfdist alone detected 10 outlier loci under divergent selection and 11 outlier loci under balancing selection. Outlier loci under balancing selection were not investigated in this study. All 10 outlier loci under divergent selection were consistently identified by BayeScan, which alone identified 26 outliers. LD analysis found that 8%–15% of possible pairwise combinations of outlier loci were statistically linked (Randomization test, *p* < .01). The proportions of significant pairs were higher than expected by chance; however, there was no locus pair that was consistently in disequilibrium in multiple populations. Total genetic variation (*H_t_*) was lower at neutral loci than at non‐neutral loci and the same trend occurred in mean genetic variation within sites (*H_w_*; Table 2). Mean global *F*_ST_ among all sites for all AFLP loci was 0.029 (*p* < .01; AMOVA). When measured using neutral or non‐neutral loci, we found global *F*_ST_ values of 0.021 (*p* < .01) and 0.039 (*p* < .01), respectively (Table 2).

### 3.3 Detection of adaptive divergence

**FIGURE 3:**
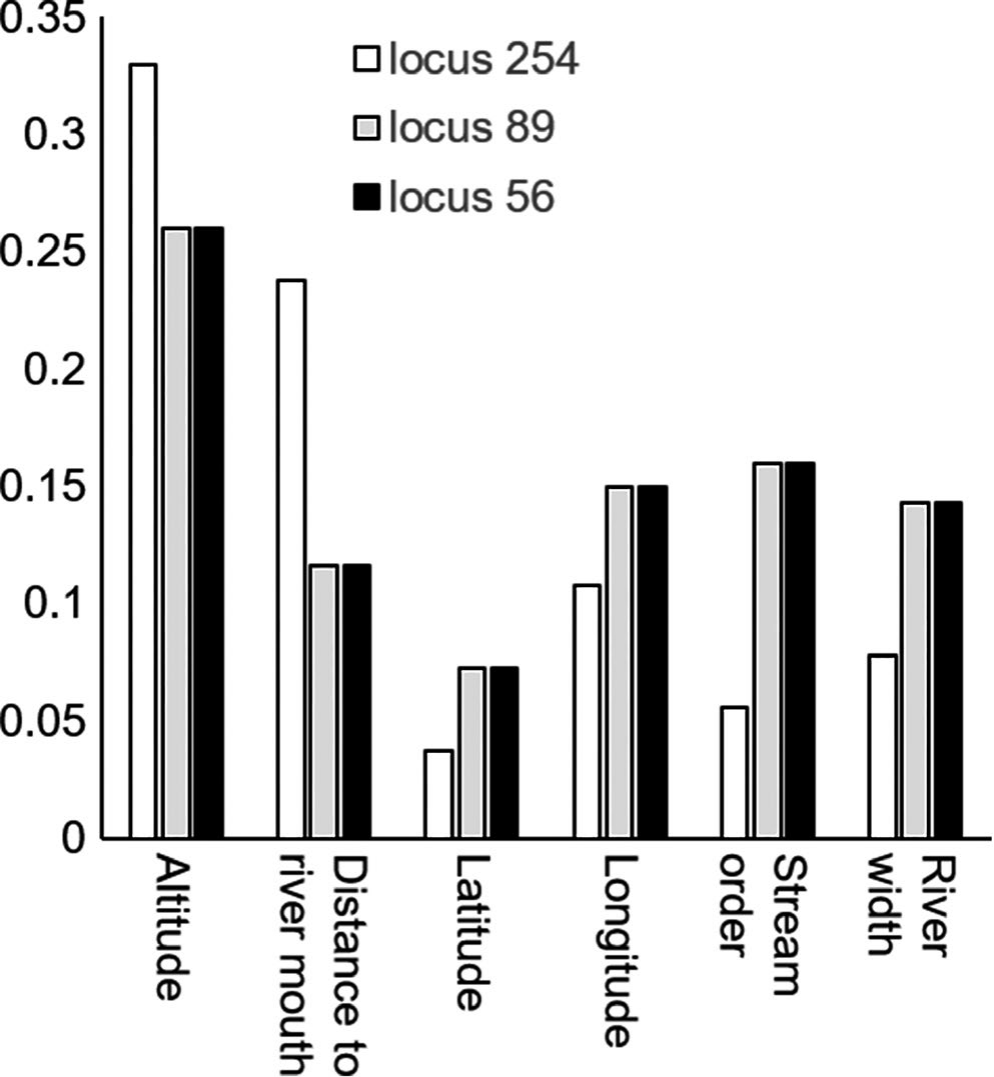
Relative importance of environmental variables based on the random forest model for three non‐neutral loci (56, 89, and 254)

**FIGURE 4:**
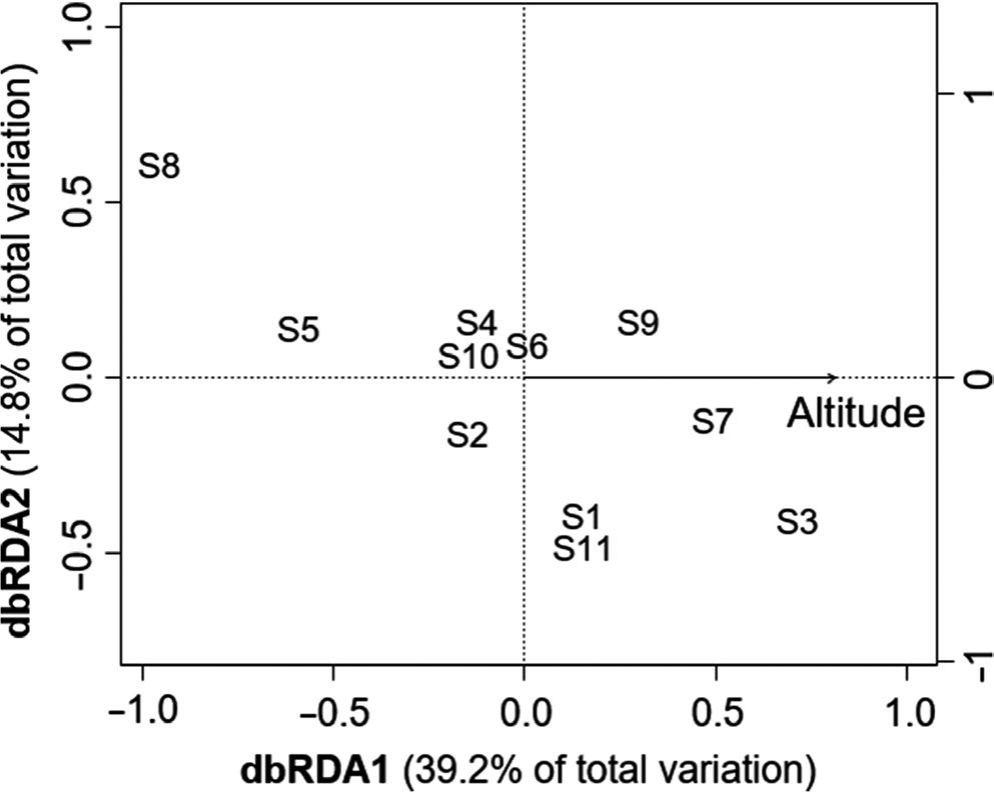
Distance‐based redundancy analysis (dbRDA) describing the influence of environmental heterogeneity on genetic variation at a non‐neutral locus (254)

We separately built one RF model for each of the 10 non‐neutral loci (Table. 1). Of the 10 non‐neutral loci, loci 56, 89, and 254 were well‐predicted (i.e., AUC > 0.7) with altitude being the most important environmental variable (Figure 3), suggesting that the genetic divergence of these loci was mainly driven by altitude. Based on dbRDA, only genetic divergence at locus 254 was significantly predicted (*p* < .05) (Figure 4). Altitude explained 54% of the genetic divergence at this locus. However, for the other non‐neutral loci, no significant relationship with environmental factors was found with dbRDA (*p* > .05).

**TABLE 1:** Sample size, AUC, OOB error rates, and key factors defined by random forest for each non‐neutral locus (sample size was shown with abundant category/rare category to show data imbalance)

Locus	Sample size (*n* = 216)	AUC	OOB	Key factor
56	202/14	0.85	5.12%	Altitude
254	175/41	0.79	12.54%	Altitude
89	199/17	0.74	11.48%	Altitude
247	204/12	0.67	7.72%	River width
36	182/34	0.52	13.43%	Stream order
90	152/64	0.51	35.22%	Latitude
98	174/42	0.51	22.78%	Latitude
97	130/86	0.51	33.09%	Distance to river mouth
260	185/31	0.50	15.41%	River width
289	200/16	0.50	12.98%	River width

**FIGURE 5:**
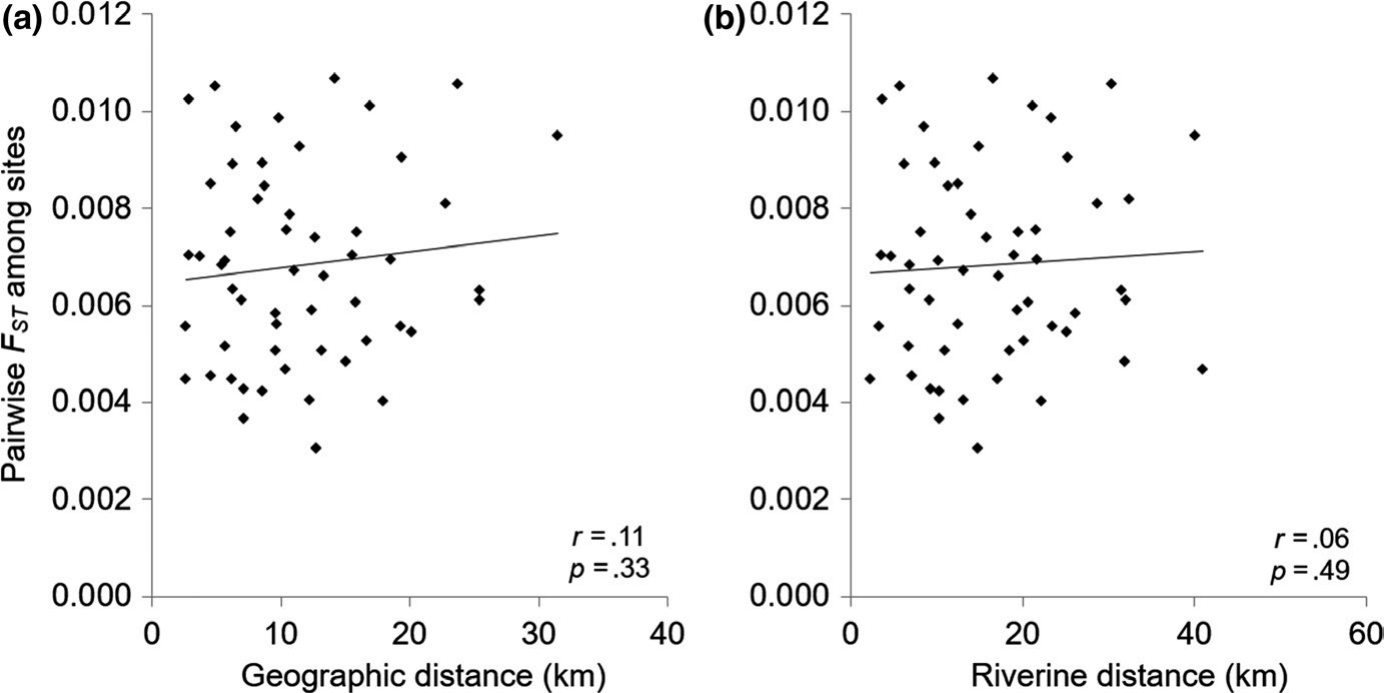
Isolation by distance calculated using geographic (a) and riverine (b) distance. Solid lines indicate correlations between Wright's fixation index (*F*ST) and geographic (*r* = .11, *p* = .33) or riverine distance (*r* = .06, *p* = .49) calculated with the Mantel tests

**FIGURE 6:**
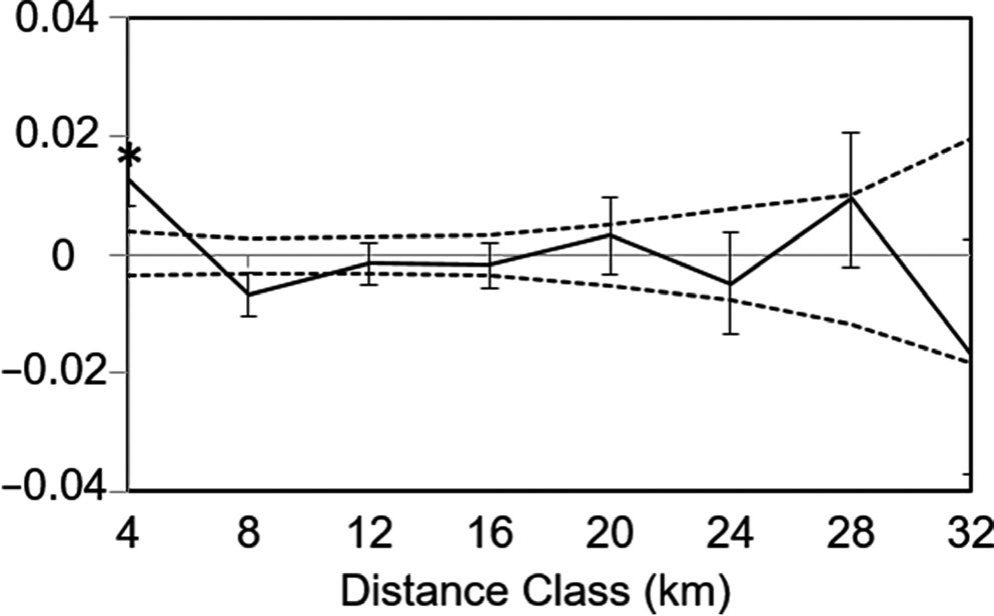
Spatial autocorrelation at 4‐km distance classes based on geographic distance for neutral loci. Dashed lines indicate permutated 95% confidence intervals, and error bars indicate jackknifed 95% confidence intervals. * indicates significant spatial autocorrelation (*p* < .05)

IBD was not significant for either geographic (*r* = .11, *p* = .33) or riverine distance (*r* = .06, *p* = .49) (Figure 5). The results of the spatial autocorrelation analysis based on neutral loci showed significant positive autocorrelation coefficients at the shortest range of 0–4 km (Figure 6).

## 4 DISCUSSION

In this study, we newly employed a modified RF model to examine the relationship between environmental factors and adaptive divergence at non‐neutral loci. An oversampling process was added using SMOTE in DMwR package in R to balance the data set before RF model building. Ordinary statistical tests of multiple linear regression method require assumptions that data are normally distributed with homogeneity of variance and independent from one another (Vittinghoff Glidden, & Mcculloch, 2012), which are often difficult to fulfill. The environmental factors investigated in this study did not show strong independency among variables (data not shown). However, the utilization of RF could overcome such difficulty, accommodating pronounced nonlinearities in the exploration of gene–environment relationships in large genomic data sets (Biau & Scornet, 2016; Breiman, 2001; Fitzpatrick & Keller, 2015).

We developed 10 RF models for each of the 10 non‐neutral loci detected by both BayeScan and Dfdist. As a result, 3 out of the 10 non‐neutral loci (loci 56, 89, and 254) showed good model prediction performance (AUC > 0.7), whereas the other 7 non‐neutral loci could not be well‐modeled. The reason why we could not build a good model for the 7 non‐neutral loci is probably because the natural selection on these loci was driven by the other environmental factors which were reported in other studies but not included in our analysis (e.g., velocity, chl‐a) (Brouwer, Bessee‐Lototskaya, ter Braak, Kraak, & Verdonschot, 2017; Li et al., 2016; Watanabe et al., 2014). Because RF can effectively perform well with a large number of variables (Genuer, Poggi, & Tuleau‐Malot, 2010), it is recommended in future studies to include as many environmental variables as possible to gain a deeper insight into the role of these factors to adaptive divergence.

To compare the performance of RF with ordinary statistical analysis, we also conducted dbRDA on each of the 10 non‐neutral loci. One locus (locus 254) was well‐modeled by dbRDA. This locus was one of the 3 loci modeled by RF, and the selected environmental factor (i.e., altitude) was consistent with RF. The low number of loci modeled in dbRDA may be because of its limited usage for testing only linear independence and its low independency. The ranking of variable importance in RF stems from the idea that if the variable is not important, then rearranging its values should not affect the prediction accuracy of the model (Breiman, 2001). This algorithm could reduce the influence of variable dependency as compared with dbRDA (Archer & Kimes, 2008; Genuer et al., 2010).

In this study, we used populations delineated by a hierarchal STRUCTURE analysis for the identification of non‐neutral loci as an alternative to geographic or phenotypic populations which are usually used in the ordinal analysis of genome scanning. The STRUCTURE analysis successfully delineated populations with significant difference in genetic terms, which is difficult to detect using visible characters such as phenotypes, ecotypes, or geographic localities (Pritchard et al., 2000). The STRUCTURE analysis can delineate genetic populations among individuals prior to any observable phenotypic divergence, and hence, may provide a means to look at early stages of adaptive divergence prior to any phenotypic divergence in the population delineation and detection of non‐neutral loci (Whiteley et al., 2011).

The hierarchical approach which we newly introduced to the STRUCTURE analysis enabled us to study the finer population structure (i.e., higher *K*) than ordinal STRUCTURE analysis, which stops the analysis once the uppermost hierarchical level is found. The number of populations (*K*) is an important determinant in the outlier detection (Foll & Gaggiotti, 2008). We also conducted outlier loci detection based on the geographic populations and the uppermost hierarchical level of STRUCTURE analysis that delineated only two populations; however, we could not detect any outlier loci. Nevertheless, fine hierarchical level (e.g., the 4th iteration in our hierarchical STRUCTURE analysis) will define weak population structure based on very subtle differences, which may introduce the risk of overfitting.

By employing a genome scan approach in this study, we comparatively used neutral and non‐neutral loci in examining genetic diversity and genetic distance. We found an interesting pattern of greater genetic divergence at non‐neutral loci than that at neutral loci. This pattern is consistent with three caddis flies species and one mayfly species studied in the same catchment system (Watanabe et al., 2014). Moreover, there are several other supporting studies which compared levels of genetic divergence between morphological traits as analogous to non‐neutral markers and neutral DNA markers in other macroinvertebrate species such as snails (Cook, 1992), spiders (Gillespie & Oxford, 1998), and damselflies (Wong, Smith, & Forbes, 2003). Based on the results of Dfdist, all the 10 non‐neutral loci were under divergent selection rather than stabilizing selection; therefore, they presented greater genetic divergence than neutral loci (Table 2).

**TABLE 2:** Genetic diversity and divergence measured using the following: (1) all loci, (2) only neutral loci, and (3) only non‐neutral loci

	*H_t_*	*H_w_*	*F*_ST_
All loci	0.1358	0.1357	0.029
Neutral loci	0.1173	0.1155	0.021
Non‐neutral loci	0.4379	0.3523	0.039

Note

*H_t_* = total expected heterozygosity; *H_w_* = mean expected heterozygosity within sites; and *F*_ST_ = Wright's fixation index among sites.

One of the most interesting findings of this study is that mountain burrowing mayfly *E. strigata* present adaptive divergence along an altitude gradient. Altitude is often reported to be closely related to a number of environmental factors that greatly influence the life cycle or development of organisms (Lytle & Poff, 2004; Múrria, Bonada, Arnedo, Prat, & Vogler, 2013; Halbritter et al., 2015). For example, altitude influences the phenology of insects, restricting the mating period to only a few days, leading to asynchronous emergence that may act as a reproductive barrier between populations (e.g., Watanabe & Monaghan, 2017; Yaegashi et al., 2014) or as a regulation of their metabolism (Gamboa, Tsuchiya, Matsumoto, Iwata, & Watanabe, 2017). This variable also influences air density, and in addition to its significance for respiration, this implies more energy is required for flight. The hemoglobin gene and other genes with a potential role for adaptation to low O_2_ may show divergence between the populations along an altitude gradient (Keller et al., 2013).

In principle, unlike non‐neutral markers, neutral markers are suitable for examining neutral process occurring under the drift–migration balance. Former population genetic studies that inferred dispersal pattern of stream insects usually used all DNA markers without classification of neutral and non‐neutral loci (Mila, Carranza, Guillaume, & Clobert, 2010; Miller, Blinn, & Keim, 2002). This may potentially cause overestimation of genetic drift because non‐neutral loci under divergent selection will increase the genetic divergence which had not occurred from genetic drift (Kirk & Freeland, 2011). Therefore, we used only neutral markers in inferring the dispersal pattern.

In the result of IBD analysis based on neutral loci, we did not observe any significant IBD for either geographic or riverine distances, suggesting that populations are not in a genetic drift–migration equilibrium at the geographic scale (Figure 5). The results of spatial autocorrelation analysis based on neutral loci showed significant positive autocorrelation coefficients at the shortest distance range (i.e., 0–4 km, Figure 6a), indicating low‐dispersal ability in this species. Such observation is understandable because mayflies are generally considered as having low‐dispersal ability in mountain streams (Barber‐James, Gattolliat, Sartori, & Hubbard, 2007). The limited dispersal distances were also observed in stoneflies due to their poor dispersal abilities (Briers, Cariss, & Gee, 2003; Briers, Gee, Cariss, & Geoghegan, 2004). In contrast, caddis flies were frequently reported to show strong dispersal ability. Yaegashi et al. (2014) reported species *Stenopsyche marmorata* exhibited dispersal ability along stream corridors up to 12 km.

In conclusion, the modified RF approach applied in this study provides an alternative method in determining constraint environmental factors for outlier loci under selection. We found that the mountain burrowing mayfly *E. strigata* present adaptive divergence along an altitude gradient using neutral and non‐neutral methods. The hierarchical STRUCTURE analysis could help to detect finer populations and increase the power of outlier detection. One limitation in this study is that we did not include many environmental factors that may also have the chance to be constrained factors and help to improve the model performance. Assessing a larger number of non‐neutral loci or do some simulations with known constraint variable use our modified RF approach will make it more applicable. Alternatively, sequencing the detected outlier loci would provide a deeper understanding of elevational adaptation of this species. In addition, besides the research in Natori River system, a comparative study in other similar area could help to provide a more comprehensive understanding of genetic adaptive divergence of *E. strigata*.

## CONFLICT OF INTEREST

None declared.

## AUTHOR CONTRIBUTIONS

**Bin Li:** Formal analysis (lead); methodology (lead); writing–review and editing (lead). **Sakiko Yaegashi:** Data curation (lead); investigation (lead). **Thaddeus M. Carvajal:** Conceptualization (equal); methodology (equal). **Maribet Gamboa:** Conceptualization (supporting); writing–original draft (supporting); writing–review and editing (supporting). **Ming‐Chih Chiu:** Conceptualization (supporting); methodology (supporting); writing–review and editing (supporting). **Zongming Ren:** Conceptualization (supporting); writing–review and editing (equal). **Kozo Watanabe:** Conceptualization (supporting); funding acquisition (lead); project administration (lead); writing–review and editing (supporting).

## Data Availability

Data supporting the results in the paper uploaded on Dryad: https://doi.org/10.5061/dryad.hmgqnk9d0. The reviewer URL is available: https://datadryad.org/stash/share/LTdzZZxLDvEZI93auRjM_U_0YijDBpCUalCypiZNj60
